# A systematic review for the impacts of global approaches to regulating electronic nicotine products

**DOI:** 10.7189/jogh.13.04076

**Published:** 2023-08-25

**Authors:** Duo Yan, Zicheng Wang, Linnea Laestadius, Kavita Mosalpuria, Fernando A Wilson, Alice Yan, Xiaoyang Lv, Xiaotian Zhang, Soumitra S Bhuyan, Yang Wang

**Affiliations:** 1 School of Public Health, Peking University, Beijing, China; 2 China Center for Health Development Studies, Peking University, Beijing, China; 3 Joseph J. Zilber College of Public Health, University of Wisconsin – Milwaukee, Milwaukee, Wisconsin USA; 4 Matheson Center for Health Care Studies, University of Utah, Salt Lake City, Utah, USA; 5 Department of Population Health Sciences, University of Utah, Salt Lake City, Utah, USA; 6 Department of Economics, University of Utah, Salt Lake City, Utah, USA; 7 Department of Research Patient Care Service, Stanford Health Care, Palo Alto, California, USA; 8 Edward J. Bloustein School of Planning and Public Policy, Rutgers University, New Brunswick, New Jersey, USA; 9 School of Public and International Affairs, Princeton University, Princeton, New Jersey, USA

## Abstract

**Background:**

The rapid increase in electronic nicotine product (ENP) use among young people has been a global public health challenge, given the potential harm of ENPs and nicotine dependence. Many countries have recently introduced legislations to regulate ENPs, but the impacts of these policies are poorly understood. This systematic review aims to critically synthesise empirical studies on the effects of global regulations regarding ENPs on the prevalence of use, health outcomes and their determinants, using the 4A marketing mix framework (acceptability, affordability, accessibility and awareness).

**Methods:**

Following the PRISMA guideline, we searched PubMed, Embase, Scopus, Web of Science, Academic Search Complete, Business Source Complete, and APA PsycINFO databases from inception until June 14, 2022 and performed citation searches on the included studies. Reviewed literature was restricted to peer-reviewed, English-language articles. We included all pre-post and quasi-experimental studies that evaluated the impacts of e-cigarette policies on the prevalence of ENP use and other health outcomes. A modified Joanna Briggs Institute (JBI) Critical Appraisal checklist for quasi-experimental studies was used for quality assessment. Due to heterogeneity of the included studies, we conducted a narrative synthesis of evidence.

**Results:**

Of 3991 unduplicated records screened, 48 (1.2%) met the inclusion criteria, most were from high-income countries in North America and Europe and 26 studies measured self-reported ENPs use. Flavour restrictions significantly decreased youth ENP use and taxation reduced adult use; mixed results were found for the impacts of age restrictions. Indoor vaping restrictions and the European Tobacco Products Directive (TPD) did not seem to reduce ENP use based on existing studies. Changes in determinants such as sales and perceptions corroborated our conclusions. Few studies assessed the impacts of other regulations such as advertising restrictions and retail licensing requirements.

**Conclusions:**

Flavour restrictions and taxes have the strongest evidence to support effective control of ENPs, while others need powerful enforcement and meaningful penalties to ensure their effectiveness. Future research should focus on under-examined policies and differential impacts across sociodemographic characteristics and countries.

**Registration:**

PROSPERO CRD42022337361

Electronic nicotine products (ENPs), also known as electronic cigarettes (e-cigarettes), electronic nicotine delivery systems (ENDS) or vaping products, were first invented by a Chinese pharmacist in 2003, and have now become widely available after two decades across the world [1]. Over this rapid expansion, the product has been aggressively marketed as a smoking cessation aid or healthier alternative to combustible cigarettes [2], as it delivers nicotine without some of the harmful chemicals generated from tobacco burning [3]. There is, however, uncertainty about the effectiveness of the claimed method for helping people quit. A recent systematic review and meta-analysis presented that when provided as a therapeutic intervention, ENPs were more effective than nicotine replacement therapy [4], but they were associated with less quitting in adult populations when regularly used as consumer products [5].

Meanwhile, a growing body of research has uncovered the potential harm of ENPs. In addition to neurotoxic nicotine, a variety of known inhalation toxins that may damage multiple organ systems have been discovered in ENP aerosols, including carbonyl compounds, monoaromatic hydrocarbons, heavy metals, and nanoparticles [6,7]. Additionally, ENPs and e-liquids (the liquid used in e-cigarette devices) have contributed to harms from explosions and poisonings [8]. Furthermore, previous longitudinal studies have raised the concern that ENP use among youth may act as a gateway to combustible cigarette and other substance use [9].

Reported use of ENPs among adolescents and young adults has been a major policy concern and is recognised as a global public health challenge [10]. In US, prevalence of current ENP use increased from 1.5% to 27.5% among high school students during 2011-2019, and meanwhile that among middle school students increased from 0.6% to 10.5% [11,12]. Use had decreased by 2022, with current use dropping to 14.1% for high school students and 3.3% for middle school students, although changes to data collection methods and COVID-19 prevent a clear comparison over time [13]. A recent meta-analysis of data from 69 countries and territories showed that the pooled prevalences of ever and current ENP use among those younger than 20 years old reached 17.2% and 7.8%, respectively [14].

Youth ENP experimentation and dependence appear to be driven by weak regulations, youth-enticing flavours, inaccurate nicotine labelling, misleading media information, poor age verification compliance, and seductive advertising [15-18]. In 2014, parties to the World Health Organization (WHO) Framework Convention on Tobacco Control were invited to consider prohibiting or regulating ENPs [19]. The overall goals were to prevent initiation by non-smokers and youth, particularly vulnerable groups, minimise potential health risks to users and protect non-users from exposure to ENP emissions [19].

In response to this recommendation, governments have been introducing diverse policy interventions in recent years. Many of these policies are based on regulatory mechanisms and options specified in MPOWER [20], a framework proposed by the WHO to promote tobacco control, including product classifications, prohibitions, component bans, taxation, age restrictions, vape-free environments, and marketing restrictions [21,22]. As of July 2022, 109 countries or jurisdictions that regulate or ban ENPs were identified by Institute for Global Tobacco Control [23].

However, the effects of e-cigarette regulatory measures may vary considerably across populations and contexts. Besides, whether regulations adapted from traditional tobacco control strategies work well for ENPs [24] needs to be confirmed. Some causal studies did not find a statistically significant reduction in ENP use after the implementation of policy interventions initially developed for combustible tobacco, including policies to increase the minimum age of sale (Tobacco 21) [25] and the extension of clean-indoor air policies to include ENPs [26]. Evidence from an experimental marketplace study also suggests that limiting or banning ENPs might result in unintended consequences such as seeking banned products from illegal sources, which may increase health risks [27].

To our best knowledge, the growing literature on the impacts of ENP regulatory policies has not been thoroughly summarised. Therefore, we conducted a systematic review to do so, in order to inform policy-making process and future research directions concerning ENP regulations. Previous reviews have identified and classified global ENP regulations into various domains [22,28], but the mechanisms by which policies affect consumers have seldomly been considered. Thus, we adopted the 4A marketing framework, which has been widely used in social marketing [29], to help readers better understand the mechanisms through which policies are intended to work. The framework was proposed by Sheth and Sisodia [30] from a consumer-value perspective, and comprises the following four constructs into which we have divided the regulations: acceptability, affordability, accessibility and awareness (**Table 1**). The aim of this review was to synthesise the studies on the effects of e-cigarette regulatory policies under the 4A marketing framework on ENPs use, health outcomes, and other related determinants such as sales, perceptions, and exposure to marketing.

**Table 1:** Classification of electronic nicotine product (ENP) policies according to 4As framework

Domain / explanation	Policies	Mechanism[31]	General descriptions
Acceptability: meeting or exceeding customers’ needs for a product / service	Flavour restrictions	Eliminate the appeal of flavored tobacco products to adolescents and young adults to prevent youth initiation	Prohibit all flavors other than tobacco and menthol in cartridge- or pod- products; or restrict flavors to adult-only stores
	Indoor vaping restrictions	Prevent renormalisation of smoking behaviors due to ENPs; reduce the convenience of vaping products	Ban ENP aerosol wherever cigarette smoke is banned, generally in indoor public places
Affordability: customers’ willingness to pay a given price for the product or service	Taxation	Increase the price of ENPs to make them more expensive	Apply excise tax to ENPs / raise taxes on ENPs
Accessibility: customers’ ability to acquire and use the product / service in two dimensions: availability and convenience	Age restrictions	Fortify the perceived difficulty in accessing ENPs among adolescents; delay youth initiation	Ban ENP sales to minors; raise the minimum age of sales to 21
	Retail licensing	Reduce youth access and availability to ENPs; reduce youth exposure to products and ads	Require that retailers be licensed to sell ENPs
	Comprehensive ban	Block legal access to ENPs	Ban the sales of all kinds of ENPs, temporarily or permanently
Awareness: product knowledge and brand awareness	Advertising	Stop spread of false or misleading health claims; reduce youth exposure to vape advertisements	Prohibit or regulate advertising, promotion, or sponsorship of ENPs
	Packaging	Enhance awareness of adverse health outcomes; prevent youth initiation and child poisoning	Require child safety packaging and nicotine warning message
Others	E-liquid regulations	Reduce addiction and prevent serious adverse consequences	Regulate nicotine concentration; prohibit use of harmful ingredients in liquid

ENP – electronic nicotine product

## METHODS

This review followed the Preferred Reporting Items for Systematic Reviews and Meta-analyses (PRISMA) [32] reporting guideline (Table S1 in the **Online Supplementary Document**), with its protocol registered in PROSPERO (CRD42022337361).

### Eligibility criteria

Eligible studies had to meet all the inclusion criteria developed from the research question using the PICOS (Population, Intervention, Comparator, Outcome, Study design) framework. Our inclusion and exclusion criteria are presented in **Table 2**.

**Table 2:** Inclusion and exclusion criteria for literature

Criteria	Included	Excluded
Population	All residents or a population subgroup (e.g., students). Populations need not be humans (e.g., vape shops, manufacturers).	No restrictions
Intervention	Any regulations enacted by national/local governments to regulate the production, marketing, or sales of ENPs.	Any regulations not enacted by governments or their agencies, such as school and prison vape-free policies
Comparator	With at least one comparison or control period or group	Without a control or comparison group
Outcome measures	Studies focused on the prevalence of electronic nicotine product use (use ENPs at least once in the past 30-days), related illnesses and injuries [32], and any factors that may influence ENP use or associated injuries, such as sales, perceptions, exposure to marketing, and quality of e-liquid.	Studies focused on the unintended effects of e-cigarette policies, such as cigarette smoking rates and substance use.
Type of study	Empirical studies with pre-post and quasi-experimental designs (defined in Table S2 in the **Online Supplementary Document**), including interrupted time series (ITS), differences-in-differences (DID), synthetic control (SC), instrumental variables (IV) and regression discontinuity (RD) designs.	Non-empirical studies and cross-sectional studies
Type of publication	Peer-reviewed articles	Other types of publication, for example, editorials, comments.

ENP – electronic nicotine product

### Search strategy

We performed an electronic search in five databases: PubMed, Embase, Scopus, Web of Science, and EBSCO (including Academic Search Complete, Business Source Complete, and APA PsycINFO) on June 14, 2022, following a search strategy developed using medical subject headings and text words related to ENPs and policies (Table S3 in the **Online Supplementary Document**). The reference lists of all included studies were retrieved to identify additional research. Only peer-reviewed articles published in English were considered.

### Study selection and data extraction

After removing duplicates from all the studies exported into Endnote 20, two independent reviewers (DY and ZW) first reviewed all retrieved titles and abstracts and then full texts following the screening criteria. Any discrepancies at either stage were resolved through discussion between the two reviewers. A third author (YW) was consulted if disagreements remained unresolved.

The authors developed and piloted a standardised data extraction form including first author, year of publication, participants, country of study, study design, policy intervention, statistical analysis methods, data source, outcomes of interest, findings and funding source (Table S4 and Table S5 in the **Online Supplementary Document**). Data were manually extracted from each study by one reviewer (DY) and verified by another (ZW), with discrepancies resolved by consultation with a third reviewer.

### Data analysis and quality assessment

Because the heterogeneous nature of studies precluded a quantitative meta-analysis, a narrative synthesis of the included empirical studies was performed to interpret and analyse their results. The 4A marketing framework was used to classify the regulations, and then the studies within each policy were categorised by outcome into the following categories, including public perception, ENP sales, prevalence of use, commercial activity, online popularity and health outcome. The results of the effects of each policy on each outcome were synthesised based on their direction (i.e., increased, decreased, and no significant change). DY and ZW independently evaluated the methodological quality of eligible studies using the modified Joanna Briggs Institute (JBI) Critical Appraisal checklist for quasi-experimental studies [33], which comprised nine items for assessing internal validity. To summarise the results of JBI appraisal, we calculated a score for each study based on how many items they satisfied (Table S6 in the **Online Supplementary Document**). Discrepancies were resolved through consultation with a third author, if necessary.

## RESULTS

### Study characteristics and quality assessment

**Figure 1:**
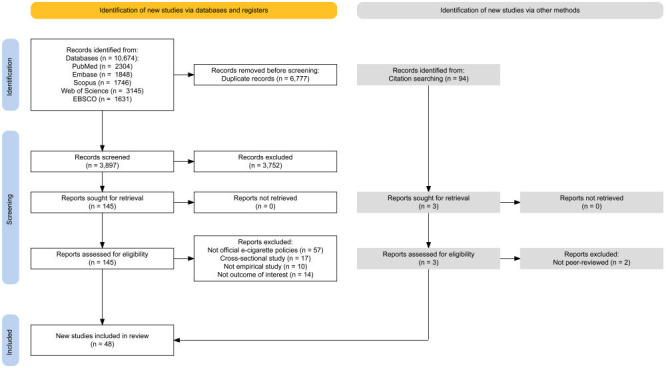
PRISMA 2020 flow diagram for study selection.

As shown in **Figure 1**, we identified 48 relevant peer-reviewed articles and summarised their descriptive information in Table S4 in the **Online Supplementary Document**. Most studies were from US (n = 31), 11 from European countries, four from Canada, and two from Asia. Except for one study [34], all were published in 2019 or later. The pre-post design was the most frequently used design (n = 20), followed by differences-in-differences (DID) (n = 19), ITS (n = 8) and instrumental variable (n = 1). Many of them lacked comparisons (n = 22) and multiple measurements of outcomes (n = 27). The quality score ranged from 5 to 9, with an average of 7.36 (see Table S6 in the **Online Supplementary Document**).

Twelve of these studies investigated impacts of ENP flavour restrictions, followed by minimum age restrictions (n = 10), the European Tobacco Products Directive (TPD) (n = 10), excise taxes (n = 8), indoor vaping restrictions (n = 5), advertising restrictions (n = 4), comprehensive bans (n = 3), packaging (n = 1), and licensing (n = 1).

Besides self-reported ENP use (n = 26), the included studies assessed the changes in perceptions (n = 6), availability (n = 4), sales (n = 4), and advertisements (n = 3) after the policy implementation. Among the 26 studies measuring ENP use, students were most frequently examined (n = 14), followed by adults (n = 9), and studies of both adolescents and adults (n = 3). Three studies examined vaping among smokers and one examined only people who vape. **Table 3** summarises the results of the included studies, with details presented below.

**Table 3:** Summary of results of the included studies by regulation type and outcome*

Policy interventions	Number of studies by effectiveness (positive / negative / mixed)	
	Current use	Other outcome
Flavour restrictions	4 / 0 / 1	8 / 0 / 0
Indoor vaping restrictions	1 / 4 / 0	-
Taxation	3 / 1 / 0	2 / 2 / 0
Comprehensive bans	-	2 / 1 / 0
Age restrictions	4 / 5 / 1	1 / 0 / 1
Licensing requirements	1 / 0 / 0	-
TPD	0 / 2 / 0	7 / 0 / 2
Advertising restrictions	-	3 / 1 / 0
Packaging	-	0 / 1 / 0

TPD – the Tobacco Products Directive

*This table shows the number of studies that supported the effects / did not support the effects / showed mixed results for the effects of policies on reducing electronic nicotine product (ENPs) use or preventing harms from ENPs.

### Acceptability

#### Flavour restrictions

##### Perception

Two studies using pre-post design applied sentiment analysis and topic modelling to examining the changes in Twitter users’ attitudes toward ENPs and policies. One analysed geocoded data during June-December 2019, and revealed that after the approval of the New York State flavour ban, the proportions of negative tweets about ENPs increased in both New York and other states, with no significant changes in attitudes toward the policy [35]. The other [36] reported a similar increase in US tweets with negative sentiment toward ENPs in response to the FDA flavour enforcement policy; meanwhile, public attitudes toward the flavour ban have become more positive.

##### ENP sales

A DID analysis [37] of 2019-2020 data suggested that Rhode Island, Washington and New York restrictions on non-tobacco-flavoured ENP sales were associated with significant reductions in total ENP sales, ranging from 25.0% to 31.3%, compared with 35 control states, though sales of tobacco-flavoured ENPs increased by over 40%.

##### ENP use

Four school-based studies [25,38-40] evaluated the impacts of flavoured tobacco restrictions on ENP use. Three Massachusetts studies [25,38,40] using DID analysis showed that restricting the sale of flavoured products to adult-only stores reduced its use among high school students [25,38], although the six-month effect of the restriction was not significant (*P* > 0.05) [40]. One study also found a decrease in non-flavoured ENP use after the regulations [38]. Similarly, a Minnesota study adopting pre-post design found that the increase in ENP use was much lower in cities restricting flavours to minors than the rest of the state [39].

Another study [41] quantified changes in vapers’ preferences toward product devices and flavours instead of actual use. It examined the data from the International Control Policy Evaluation Project (ITC) Youth Tobacco and Vaping Survey [41] and found that disposable e-cigarettes exempt from US flavour restrictions increased to a greater extent among youth vapers in the US, as compared to Canada and England without the regulation. However, no significant differences were observed among US vapers in the proportion of those who usually used restricted flavours before and after the implementation.

##### Commercial activity

Two cohorts [42,43] and one repeated cross-sectional [44] study, examining the change in availability of flavoured vaping products associated with county-wide restrictions, consistently found significant decreases. Two studies determined that flavoured vaping product availability in Massachusetts retailers significantly decreased following the implementation of local flavour restrictions [42,43]. Similarly, a California survey [44] on retailers located in Alameda and San Francisco Counties found that the availability of all vaping products either decreased or remained stable from 2015 to 2020 in cities which enacted flavoured (including menthol) tobacco sales restrictions, while the availability of ENPs (excluding vape pens and menthol e-cigarettes) significantly increased in control cities. Moreover, interior advertising frequency for ENPs dropped in cities with the restrictions while it increased in control cities.

##### Online popularity

An ITS analysis [45] of the relative search volumes (RSV) of vaping products found that the RSV for Juul decreased sharply after the FDA banned cartridge-based e-cigarettes in flavours other than tobacco and menthol in 2020, while the RSV for Puff Bar (a disposable vaping product) accelerated in contrast.

#### Indoor vaping restrictions

##### ENP use

Four nationally representative, US [26,46,47] and Canada [48], and one Massachusetts-based survey study [25] used DID designs to evaluate the impacts of adding ENPs into indoor smoke-free policy on vaping. Four [25,26,46,48] of them reported no significant relationship between the restrictions and ENP use. One study found that among grade 9-12 students from 34 US states [47], prevalence of ever and past 30-day ENP use in states with ENP-inclusive smoke-free policies decreased during 2017-2019, while it increased in states without the policies.

### Affordability

#### Taxation

##### ENP sales

Three studies assessed the impact of taxation on ENP sales in convenience stores by analysing the NielsenIQ Retail Scanner Data set. Amato [34] performed a trend analysis using Joinpoint regressions, finding a significant increase in Minnesota’s ENP sales immediately after imposing the taxes; however, the total sales dropped significantly below expected values after eight weeks. Similarly, IV analyses on data from 51 US localities estimated that a 1.00 US dollars (US$) tax increase raised ENP prices by US$0.90 and reduced weekly e-liquid sales by 919 millilitres (ml) per 100 000 adult residents [49]. Nevertheless, there were no significant changes in ENP sales at convenience stores in the Greater Boston area after a 75% excise tax on nicotine-containing ENPs was imposed [50].

##### ENP use

Among four studies [47,51-53] that examined the effect of taxation on ENP use, three adult cohorts [51-53] supported that excise tax decreased current use while one school-based sample [47] did not. The 2011-2018 Behavioral Risk Factor Surveillance System and the National Health Interview Survey data [51] found a US$1.00 increase in tax per fluid ml of e-liquid was estimated to reduce the probabilities of current and daily vaping by 0.5 and 0.2 percentage points, respectively. Another DID analysis [52] of the Tobacco Use Supplement to the Current Population Survey showed that respondents living in states with an excise tax policy had a significantly lower likelihood of ENDS use. In an Indonesia cohort [53], adult smokers and vapers reported an average 0.5-day decrease in the number of days when they used ENPs in the past week, and the proportion of daily users also decreased (75.9% to 63.6%) after a 57% ad-valorem tax on e-liquids was approved. However, the state-wide taxes had no significant effect on ENP use reductions among US 9-12 graders [47].

##### Commercial activity

One trend analysis of Yelp data [54] reported that the number of listed vape shops in Pennsylvania increased by 23% in less than 2 years after imposing a 40% tax on both ENDS and e-liquids.

### Accessibility

#### Comprehensive bans

##### ENP sales

Two articles [37,50] considered Massachusetts’ three-month emergency ban on all vaping-related products in September 2019, and demonstrated its effects on reducing ENP sales. An ITS analysis [50] reported that the Greater Boston area saw a significant decrease in ENP sales per capita after the state ban; however, the trend of the sales reversed following the ban lifting. Similarly, a DID analysis [37] of Information Resources, Inc. data presented that prohibition of all ENPs in Massachusetts was associated with a 94.4% (95% confidence interval (CI) = 93.4%-95.2%) reduction in four-week total unit sales in November, 2019, compared with 35 control states without restrictions.

##### Commercial activity

An Indian pre-post design study [55] examined the regulation compliance of online retailers after banning all types of ENPs in 2018, finding that all websites which sold ENPs before the legislation were still selling the products and accessories without any age verifications.

#### Age restrictions

##### Perception

Of ten articles that investigated the impacts of the minimum legal purchasing age, two [56,57] measured changes in harm perception towards ENPs and their use, attributable to bans on sales to minors, while the remainder [25,47,58-63] published in 2021 or later focused on the effects of Tobacco 21 raising the minimum tobacco sales age to 21 on youth use.

One Canadian study using a DID method [57] reported that youths in provinces with a ban were 18% less likely to believe that e-cigarettes posed no harm to their health. However, an annual cross-sectional survey of Floridian 6-12 graders [56] showed that more youths perceived that ENDS were less harmful than cigarettes, not as addictive as cocaine or heroin, and easy to quit despite the minimum age policy implementation.

##### ENP use

The ten studies [25,47,56-63] on the association between current ENP use and minimum age restrictions have yielded mixed results. Using a triple differences method, a Canadian study found that the increase in prevalence of youth ENP use from 2013 to 2017 in provinces with age restrictions was 3.1 percentage points lower (95% CI = 0.2-6.0) than that in control provinces [57]. However, Florida’s minimum-age policy failed to significantly reduce current ENDS use among high and middle school students [56]. Regarding Tobacco 21, three studies uncovered significant decreases in use among adolescents in multiple US states due to the policies [47,58,63], while two studies using data from California Healthy Kids Survey found that Tobacco 21 was associated with increases in current ENP use [59,61], and no relationship was found from the 2011-2017 Massachusetts Youth Health Survey [25]. As for young adults, one cohort showed ENP use slightly decreased among Southern California youth after the policy implementation, while another found use sharply increased in Columbus [60,62].

#### Retail licensing

##### ENP use

Only one US study [64] assessed the extent to which ENP retail licensing influenced youth use. Analysing data from Youth Risk Behavior Surveillance System, this DID study found that retail licensing requirement in Pennsylvania reduced ENP use among adolescents by 21.6% and 30.7%, respectively, when compared with control states New York and Virginia.

### Awareness

#### TPD

The TPD required all the European Union member states to introduce the regulations by May 2016 [65], including limitations on nicotine concentration of e-liquids, child-resistant packaging, health warnings on packaging, and a ban on advertising and promotion.

##### Perception

One study [66] using data from yearly surveys (2015-2017) of the ITC Netherlands Survey reported that vapers showed increased perceptions of ENP’s addictiveness and toxicity than cigarette-only smokers. Another study [67] found no consistent patterns for significant changes in smokers’ support for ENP policies across seven European countries over two years after TPD implementation.

##### ENP use

Four survey studies [68-71] investigated the changes of ENP use in response to TPD implementation and consistently found no significant relationship between the regulations and use reductions. The proportions of current users in Finish adolescents and adults remained low at approximately 2% during 2014-2018 [71]. The prevalence of regular (at least weekly) ENP use in Wales students marginally increased from 4.2% in 2015 to 4.8% in 2017; but it doubled in England students from 1.7% in 2014 to 3.4% in 2016 [68]. Likewise, two cohort studies of smokers [69,70] found little increases in proportion of those who reported currently ENP use after TPD was introduced, suggesting limited effects of the policy.

##### Commercial activity

Three studies on ITC Four Country Smoking and Vaping Surveys showed that more European e-cigarette users noticed the warning labels and leaflets required by TPD [66,70,72]. The percentages of respondents in ITC projects who reported having been exposed to ENP advertising, promotion or sponsorship increased in Germany, Greece, Hungary and Spain during 2016-2018, while decreases were observed in both Poland and Romania [73]. An analysis of the composition of e-liquids [74] revealed that although not all manufacturers managed to produce and label their products accurately, nicotine labelling discrepancies were decreasing gradually. Another pre-post design study [75] demonstrated that the compliance of e-cigarette refill liquids with TPD regulations on labelling, packaging and technical designs had improved in nine European countries.

#### Advertising restrictions

##### Commercial activity

Four studies [76-79] assessed the impact of ENP advertising restrictions. After Ontario banned vape advertising by retailers, Martin et al. [77] observed a 78.2% reduction of vape advertisements within 800 m of secondary schools. After Poland banned all advertising, promotion and sponsorships in 2016 [78], one pre-post study showed that the presence of any ENP advertising at point-of-sale significantly decreased from 92.0% in 2014 to 30.0% in 2019. A DID study of Canadian youth aged 16-19 [76] revealed that the increase in their likelihood of often noticing promotions during 2017-2019 was substantially lower in provinces with stricter restrictions. Additionally, Laestadius [79] evaluated the impact of FDA rules requiring addiction warnings to be placed on advertising for e-liquids containing nicotine, finding that 36.4% of US Instagram advertising posts displayed warnings.

#### Packaging

##### Health outcome

One ITS analysis [80] showed that the reduction in poisoning cases resulting from nicotine exposure involving ENPs was not statistically significant after implementation of the American Child Nicotine Poisoning Prevention Act, which required liquid nicotine containers used in open-tank systems to be specially packaged to prevent children under the age of fivr years from accessing their contents.

## DISCUSSION

This systematic review is the first to comprehensively examine empirical studies on the impacts of ENP policies on behaviours and market outcomes across countries. The literature search identified nine policy areas divided into four domains specified in the 4A marketing strategy. Our findings support that limiting access to flavours for minors and reducing affordability for adults are effective in reducing ENP use. Limited evidence has found that taxation might be ineffective in reducing adolescent ENP use. We also found mixed results for the effects of age restrictions on ENP use; nonetheless, indoor vaping restrictions and the TPD were repeatedly shown not to be associated with a reduction in ENP use. Only one study focused on health outcomes and investigated the impact of ENP regulations on e-cigarette related poisoning events. Changes in harm perceptions, sales, availability, and other ENP use determinants corroborated our conclusions. Evidence of relationships between ENP use and other included policies was limited.

Contrary to prior findings of positive impacts of smoke-free legislation on cigarette use [81,82], one of two adolescent and all adult studies reported that indoor vape-free laws did not result in a statistically significant decline in ENP use. This might imply laxity in local enforcement resulting in poor compliance with vaping restrictions in public places [83] and people’s tolerance to others’ vaping [84]. Such contrasting observations can be explained by “stealth vaping,” or discreet vaping in prohibited places. It has been reported as the preferred way of vaping by approximately two-thirds of adult vapers in an online survey [85]. Similarly, second hand e-cigarette vapors are perceived as less harmful than combustible cigarettes by the public [86], which may explain why few vapers encounter resistance to their vaping behaviour in smoke-free places [84]. Alternatively, many established e-cigarette users primarily vape in their cars, homes, and other non-public locations [87], which are not subject to the law. Taxation and flavour restrictions may be more effective than indoor vape-free policies in deterring vaping.

Regarding the mixed results for age restrictions, one possible reason is that compliance varies from place to place [88]. As mentioned in results, T21 policy implemented in California was not effective as in other states, and high rates of illicit sales to minors might be the cause. Of California tobacco and vape shops, 49.8% failed to check ID for underage decoys who attempted to buy vaping products in 2018 [89]. Meanwhile, minors may also show retailers fake IDs similar to tactics for purchasing alcoholic beverages [90], or acquire ENPs from an adult friend [91] to circumvent the access restriction. Online purchasing without age verifications also posed a new challenge for the regulations as it has become the predominant way of acquiring ENDS among adolescents and youth since the COVID-19 pandemic [17]. Besides, urban population and non-minorities were generally more likely to benefit from age restrictions [58,59], increasing concerns about disparities in the use of ENPs.

Unlike other policies that directly restrict access to electronic nicotine products or flavours, Article 20 of the TPD has established European industry standards for ENPs and required health warnings to raise public awareness about harmful effects of ENPs. Although population surveys [70] and chemical characterisation of e-liquids [73] have shown that the TPD has partially achieved the anticipated effects in Europe, a multi-country survey found that the proportion of vapers who noticed health warnings and leaflets required by the TPD was still very low [66,70,72]. In 2018, only 3.8% and 11.6% of vapers in the past 30 days noticed the warning labels and leaflets of ENP, respectively, with more than half of them having never read the information [70]. This may explain why a decline in ENP current use did not occur after the regulation implementation. Meanwhile, the lack of an independent control group and the limited number of studies identified may result in the failure to observe reductions in vaping rates.

### Implications for practitioners and policymakers

Our findings shed light on developing targeted policy interventions and improving regulation enforcement to prevent ENP initiation. The concern about violations of regulations is not unique to specific regions or limited to certain policies, emphasising the importance of manufacturer, distributor, and retailer accountability. For example, six months after San Francisco passed a comprehensive prohibition on sales of flavoured tobacco products in April 2018, only 17% of inspected stores confirmed no flavoured tobacco [92]. However, after the formal execution by Department of Public Health, the compliance rate jumped to 80% [92], indicating the necessity of enforcement and monitoring. Meanwhile, differential effects of regulations across sociodemographic characteristics urge policymakers to design tailored interventions to reduce health disparities, which requires a comprehensive consideration of numerous factors including cultural context, advertising exposure, stigmatisation and enforcement of laws [93]. In addition, policy impacts would be improved by educating tobacco retailers, youth, parents, as well as creating changes in social environments (e.g., media and online publicity), especially in areas currently without strong regulatory power.

Given that prior studies have overwhelmingly supported that e-cigarettes and cigarettes are economic substitutes [94-96], another worry is that the implementation of legislation that only targets ENP products, including taxation [53] and vape-free laws [46], may increase combustible cigarette use and sales. Pesko et al. [51] estimated that a US$1.00 increase in tax per vaping fluid ml would increase daily smoking propensity by 0.6 percentage points (ppts) among US adults. Friedman et al. [46] reported that adding vaping restrictions to smoke-free workplace policies did not reduce current vaping, but neutralised more than half of the association between the original smoke-free workplace policies and current smoking. Moreover, the unintended effects may disproportionately impact vulnerable groups [58,59]. For example, using United States birth records between 2010 and 2016, a panel data analysis showed that county-level ENP indoor vaping restrictions increased within-pregnancy smoking among pregnant teenagers by 0.2 ppts. This was mainly due to a 0.6 ppt increase in rural areas [97]. Besides, some researchers are concerned that although a flavour restriction would reduce vaping rates, it would also decrease the likelihood of smokers using ENPs, which is considered as a safer way to obtain nicotine [98]. Therefore, policymakers should be aware of the potential for vapers to transition to smoking as a result of regulations. They should adopt additional policies to prevent an increase in the number of smokers. For example, all smokers who wish to quit may be eligible for free clinical smoking cessation services, including nicotine replacement therapy, counselling, and pharmacotherapy [99].

### Limitations of evidence included and implications for future research

The studies included in our review have some limitations, with most suffering from selection bias. Response rates of some surveys were lower than 70% [25,39], without detailing characteristics of non-response populations. The studies of adolescents largely used public school samples, limiting generalisability to adolescents who left school and private school students. ENP sales data from Nielsen do not capture vape shop or online sales while approximately 70% US vapers buy products online or from vape shops [100]. Outcome measurements were also often subject to participants’ possible inaccurate recollection of ENP related experiences in the past 30 days, such as use, exposure to marketing, and noticing warning labels.

Methodological flaws revealed by our review should be accounted in interpreting the results. Simple pre-post designs adopted in 20 studies failed to preclude the trend effects of growing prevalence of ENP use in recent years regardless of regulations being implemented, compromising causal inferences. Given availability of data, it is also almost impossible to precisely measure the impact of a single policy when more than one of them are introduced in a short period and overlapped; for instance, in Pennsylvania, retail licensing requirements for ENDS went into effect in July 2016, and just three months later, a 40% tax was imposed on ENDS and e-liquids [54] in the state. Likewise, tobacco control policies are usually implemented at the county or city level, but some studies aggregated them to the state level due to data limitations [26,41,45,47]. In this case, misclassifying individuals into areas with or without the implementation of the policies may underestimate the effects.

We have identified research gaps to be addressed in future studies. First, the lack of studies on the impact of flavour restrictions on adults, impact of taxes on minors, as well as mixed results regarding vape-free policies and age restrictions call for further investigations. Second, given geographic variations in impacts of the same policies, more research is needed to advance our understanding of potential social and cultural influencing factors across countries / states. Third, few studies have considered the disproportionate effects of the policies on vulnerable people [58,61] and the effectiveness of regulations on online sale of ENPs, which both warrant comprehensive examination.

### Limitations of the review process

We acknowledge several limitations of this review. First, we only searched English papers and studies identified are mostly from high-income countries in North America and Europe [14], which may limit generalisability of our findings. Second, we did not search grey literature, although a search of included references was conducted to reduce the bias of missing literature. As many insignificant results were included in our review, we are confident that publication bias did not change the overall conclusions. Third, data extraction was conducted by single reviewer and checked by another author. The lack of independence and duality in this process may introduce risk of errors to the review. Fourth, due to considerable heterogeneity regarding methodological aspects of studies, we could not quantify the magnitude of the effects of policies.

## CONCLUSIONS

Our review has contributed to informing evidence-based practice in ENP market regulations, a field where scattered evidence in its infancy has largely remained concentrated in developed countries across Europe and North America. Flavour restrictions and excise taxes appear to be the most promising strategies for reducing ENP use, corroborated by observed desirable changes in the use-related determinants in response to the implementation of regulations. Very few studies have been conducted to assess the effect of ENP regulation on related health outcomes. We also highlight concerns for practitioners regarding increases in smoking rates and health inequities triggered by the policies. Future studies are warranted to focus on health outcomes, measure the effects of under-examined policies including retail licensing and advertising restrictions, particularly in developing countries, as well as differential policy impacts across sociodemographic characteristics.

## Additional material


Online Supplementary Document
