# Supplementary material for: A systematic review for the impacts of global approaches to regulating electronic nicotine products
Source: J Glob Health. 2023 Aug 25;13:04076. doi: 10.7189/jogh.13.04076 (PMC10451104; doi:10.7189/jogh.13.04076)
Supplement: Online Supplementary Document [file jogh-13-04076-s001.pdf]

Supplementary Material

Article title: A Systematic Review for the Impacts of Global Approaches to Regulating Electronic Nicotine Products

Journal: Journal of Global Health

Table of Contents

Table S1. PRISMA 2020 checklist

Table S2. Definition of quasi-experimental designs

Table S3. Search strategies for each database

Table S4. Study characteristics

Table S5. Main findings of included studies by policy classification

Table S6. Quality assessment based on the JBI Critical Appraisal checklist for quasi-experimental studies

Table S1 PRISMA checklist

Section and Topic	Item #	Checklist item	Location where item is reported
**TITLE**			
Title	1	Identify the report as a systematic review.	1
**ABSTRACT**			
Abstract	2	See the PRISMA 2020 for Abstracts checklist.	3
**INTRODUCTION**			
Rationale	3	Describe the rationale for the review in the context of existing knowledge.	4-6
Objectives	4	Provide an explicit statement of the objective(s) or question(s) the review addresses.	6
**METHODS**			
Eligibility criteria	5	Specify the inclusion and exclusion criteria for the review and how studies were grouped for the syntheses.	7, Table 2
Information sources	6	Specify all databases, registers, websites, organisations, reference lists and other sources searched or consulted to identify studies. Specify the date when each source was last searched or consulted.	7
Search strategy	7	Present the full search strategies for all databases, registers and websites, including any filters and limits used.	7, S2
Selection process	8	Specify the methods used to decide whether a study met the inclusion criteria of the review, including how many reviewers screened each record and each report retrieved, whether they worked independently, and if applicable, details of automation tools used in the process.	7
Data collection process	9	Specify the methods used to collect data from reports, including how many reviewers collected data from each report, whether they worked independently, any processes for obtaining or confirming data from study investigators, and if applicable, details of automation tools used in the process.	7
Data items	10a	List and define all outcomes for which data were sought. Specify whether all results that were compatible with each outcome domain in each study were sought (e.g. for all measures, time points, analyses), and if not, the methods used to decide which results to collect.	Table 2
	10b	List and define all other variables for which data were sought (e.g. participant and intervention characteristics, funding sources). Describe any assumptions made about any missing or unclear information.	7, Table 2
Study risk of bias assessment	11	Specify the methods used to assess risk of bias in the included studies, including details of the tool(s) used, how many reviewers assessed each study and whether they worked independently, and if applicable, details of automation tools used in the process.	8, S6
Effect measures	12	Specify for each outcome the effect measure(s) (e.g. risk ratio, mean difference) used in the synthesis or presentation of results.	8
Synthesis methods	13a	Describe the processes used to decide which studies were eligible for each synthesis (e.g. tabulating the study intervention characteristics and comparing against the planned groups for each synthesis (item #5)).	8
	13b	Describe any methods required to prepare the data for presentation or synthesis, such as handling of missing summary statistics, or data conversions.	8
	13c	Describe any methods used to tabulate or visually display results of individual studies and syntheses.	8
	13d	Describe any methods used to synthesize results and provide a rationale for the choice(s). If meta-analysis was performed, describe the model(s), method(s) to identify the presence and extent of statistical heterogeneity, and software package(s) used.	8

Section and Topic	Item #	Checklist item	Location where item is reported
	13e	Describe any methods used to explore possible causes of heterogeneity among study results (e.g. subgroup analysis, meta-regression).	Na
	13f	Describe any sensitivity analyses conducted to assess robustness of the synthesized results.	Na
Reporting bias assessment	14	Describe any methods used to assess risk of bias due to missing results in a synthesis (arising from reporting biases).	8
Certainty assessment	15	Describe any methods used to assess certainty (or confidence) in the body of evidence for an outcome.	8, Table 3
**RESULTS**			
Study selection	16a	Describe the results of the search and selection process, from the number of records identified in the search to the number of studies included in the review, ideally using a flow diagram.	Figure 1
	16b	Cite studies that might appear to meet the inclusion criteria, but which were excluded, and explain why they were excluded.	Figure 1
Study characteristics	17	Cite each included study and present its characteristics.	S4
Risk of bias in studies	18	Present assessments of risk of bias for each included study.	S6
Results of individual studies	19	For all outcomes, present, for each study: (a) summary statistics for each group (where appropriate) and (b) an effect estimate and its precision (e.g. confidence/credible interval), ideally using structured tables or plots.	Table 3, S5
Results of syntheses	20a	For each synthesis, briefly summarise the characteristics and risk of bias among contributing studies.	8-9
	20b	Present results of all statistical syntheses conducted. If meta-analysis was done, present for each the summary estimate and its precision (e.g. confidence/credible interval) and measures of statistical heterogeneity. If comparing groups, describe the direction of the effect.	9-16
	20c	Present results of all investigations of possible causes of heterogeneity among study results.	Na
	20d	Present results of all sensitivity analyses conducted to assess the robustness of the synthesized results.	Na
Reporting biases	21	Present assessments of risk of bias due to missing results (arising from reporting biases) for each synthesis assessed.	23
Certainty of evidence	22	Present assessments of certainty (or confidence) in the body of evidence for each outcome assessed.	Table 3
**DISCUSSION**			
Discussion	23a	Provide a general interpretation of the results in the context of other evidence.	17-18
	23b	Discuss any limitations of the evidence included in the review.	20-21
	23c	Discuss any limitations of the review processes used.	21
	23d	Discuss implications of the results for practice, policy, and future research.	18-20
**OTHER INFORMATION**			
Registration and	24a	Provide registration information for the review, including register name and registration number, or state that the review was not registered.	7

Section and Topic	Item #	Checklist item	Location where item is reported
protocol	24b	Indicate where the review protocol can be accessed, or state that a protocol was not prepared.	7
	24c	Describe and explain any amendments to information provided at registration or in the protocol.	7
Support	25	Describe sources of financial or non-financial support for the review, and the role of the funders or sponsors in the review.	2
Competing interests	26	Declare any competing interests of review authors.	2
Availability of data, code and other materials	27	Report which of the following are publicly available and where they can be found: template data collection forms; data extracted from included studies; data used for all analyses; analytic code; any other materials used in the review.	2

From: Page MJ, McKenzie JE, Bossuyt PM, Boutron I, Hoffmann TC, Mulrow CD, et al. The PRISMA 2020 statement: an updated guideline for reporting systematic reviews. BMJ 2021;372:n71. doi: 10.1136/bmj.n71

For more information, visit: http://www.prisma-statement.org/

Table S2. Definition of quasi-experimental designs

Terms	Definition
**Pre-post design**	A method for assessing the effects of an intervention by comparing outcomes before and after the intervention implementation
**Difference in Differences (DID)**	A method that identifies causal effects by contrasting the change in outcomes pre- and post- intervention, for the treatment and control groups. Data may be truly longitudinal or repeated cross-sections.
**Interrupted time series (ITS)**	A method that assesses the causal effect by comparing the observed post-intervention trend to the extrapolated trend from the pre-intervention time series.
**Synthetic controls (SC)**	A method that uses a weighted combination of comparison units (the "synthetic control") to represent the counterfactual (what would have happened in the absence of the treatment.
**Regression discontinuity (RD)**	A method that estimates the treatment effect by comparing units either side of a threshold on a continuous variable which determines treatment status.
**Instrumental variables (IV)**	A method that used an exogenous variable to estimate the treatment effect using multi-stage or simultaneous equations regression analysis.

Table S3. Search strategy

Database	Search terms for each database
**PubMed**	(("Electronic Nicotine Delivery Systems"[MeSH Terms] OR "Vaping"[MeSH Terms] OR ("Electronics"[MeSH Terms] AND ("Tobacco Products"[MeSH Terms] OR "Smoking"[MeSH Terms] OR "Nicotine"[MeSH Terms])) OR ("e cig*"[Title/Abstract] OR "e cigarette*"[Title/Abstract] OR "electronic cigarette"[Title/Abstract] OR "electronic cigarettes"[Title/Abstract] OR "vape"[Title/Abstract] OR "Vaping"[Title/Abstract] OR "e liquid*"[Title/Abstract] OR ("electronic nicotine delivery system"[Title/Abstract] OR "Electronic Nicotine Delivery Systems"[Title/Abstract])) OR ("JUUL"[Title/Abstract] OR "juuling"[Title/Abstract] OR "electronic cigarette"[Other Term] OR "e cig*"[Other Term] OR "electronic nicotine delivery system"[Other Term] OR "ENDS"[Other Term] OR "Vaping"[Other Term] OR "vape"[Other Term])) AND ("Legislation as Topic"[MeSH Terms] OR "Policy"[MeSH Terms] OR "Taxes"[MeSH Terms] OR "social control, formal"[MeSH Terms] OR "legislation and jurisprudence"[MeSH Subheading] OR "law"[Title/Abstract] OR "laws"[Title/Abstract] OR "ban"[Title/Abstract] OR "bans"[Title/Abstract] OR "Policy"[Title/Abstract] OR "policies"[Title/Abstract] OR "regulation"[Title/Abstract] OR "regulations"[Title/Abstract] OR "restriction"[Title/Abstract] OR "restrictions"[Title/Abstract] OR "tax"[Title/Abstract] OR "taxation"[Title/Abstract] OR "legislate"[Title/Abstract] OR "legislation"[Title/Abstract] OR "licens*"[Title/Abstract] OR "legislation"[Other Term] OR "Policy"[Other Term] OR "policies"[Other Term] OR "tobacco control"[Other Term] OR "taxation"[Other Term] OR "law"[Other Term] OR "rule"[Other Term] OR "regulation"[Other Term] OR "ban"[Other Term] OR "licensing"[Other Term] OR "prevention"[Other Term])) NOT ("comment"[Publication Type] OR "editorial"[Publication Type] OR "letter"[Publication Type] OR "news"[Publication Type] OR "review"[Publication Type] OR "systematic review"[Publication Type] OR "meta-analysis"[Publication Type])
**Embase**	(electronic cigarette/exp OR vaping/exp OR e cig*:ti,ab,kw OR electronic cigarette:ti,ab,kw OR electronic cigarettes:ti,ab,kw OR vape:ti,ab,kw OR vaping:ti,ab,kw OR e -liquid?:ti,ab,kw OR e - cigarette*:ti,ab,kw OR juul:ti,ab,kw OR juuling:ti,ab,kw OR electronic nicotine delivery system:ti,ab,kw OR electronic nicotine delivery systems:ti,ab,kw) AND (law?:ab,ti OR ban?:ab,ti OR rule?:ab,ti OR policy:ab,ti OR policies:ab,ti OR legislat*:ab,ti OR regulation?:ab,ti OR restriction?:ab,ti OR tax:ab,ti OR taxation:ab,ti OR licens*:ab,ti OR tobacco control:kw OR prevention:kw OR law/exp OR policy/exp OR tax/exp OR social control/exp OR legislation and jurisprudence/ exp) AND ([article]/lim OR [article in press]/lim) AND [english]/lim NOT [animals]/lim
**Scopus**	(TITLE-ABS-KEY(e-cig* OR "electronic cigarette" OR "electronic cigarettes" OR vape OR vaping OR "e-liquid?" OR "e-cigarette*" OR juul OR juuling OR "electronic nicotine delivery system" OR "electronic

	nicotine delivery systems")) AND (TITLE-ABS-KEY(law? OR ban? OR rule? OR policy OR policies OR legislat* OR regulation? OR restriction? OR tax OR taxation OR licens*)) AND (LIMIT-TO (DOCTYPE,"ar") OR LIMIT-TO (DOCTYPE,"Undefined")) AND (LIMIT-TO (LANGUAGE,"English"))
**Web of Science**	((TS=(e-cig* OR "electronic cigarette" OR "electronic cigarettes" OR vape OR vaping OR "e-liquid?" OR "e-cigarette*" OR juul OR juuling OR "electronic nicotine delivery system" OR "electronic nicotine delivery systems")) AND TS=(law? OR ban? OR rule? OR policy OR policies OR legislat* OR regulation? OR restriction? OR tax OR taxation OR licens*)) AND ((DT==("ARTICLE" OR "OTHER" OR "CLINICAL TRIAL" OR "UNSPECIFIED")) NOT (SILOID==("MEDLINE")))
**EBSCO**	S1 TI ("e-cig*" OR "electronic cigarette" OR "electronic cigarettes" OR vape OR vaping OR "e-liquid?" OR "e-cigarette*" OR juul OR juuling OR "electronic nicotine delivery system" OR "electronic nicotine delivery systems") OR AB ("e-cig*" OR "electronic cigarette" OR "electronic cigarettes" OR vape OR vaping OR "e-liquid?" OR "e-cigarette*" OR juul OR juuling OR "electronic nicotine delivery system" OR "electronic nicotine delivery systems") OR SU ("electronic nicotine delivery system" OR "electronic cigarette" OR juul OR vaping)<br>S2 SU (policy OR legislation OR regulation OR taxation OR licensing) OR TI (law? OR ban? OR rule? OR policy OR policies OR legislat* OR regulation? OR restriction? OR tax OR taxation OR licens*) OR AB (law? OR ban? OR rule? OR policy OR policies OR legislat* OR regulation? OR restriction? OR tax OR taxation OR licens*)<br>S3 S1 AND S2

Table S4. Study characteristics

Study ID	Country/state	Policy classification	Policy level	Study design	Population	Outcome measure	Data source (if its a secondary analysis)	Quality score
Ali et al (2022)	US	comprehensive ban; flavor ban	state	DID	NA	e-cigarette sales	IRI	9
Amato and Boyle (2016)	US/Minnesota	taxation	state	ITS	NA	e-cigarette sales	NRSD	9
Azagba et al (2019)	US/Pennsylvania	retail licensing requirement	state	DID	students (grade 9-12)	e-cigarette use in last month	YRBSS	7
Barhdadi et al (2021)	Belgium	TPD	country	pre-post	NA	concentration of nicotine and impurities; nicotine-stability	NA	7
Choi et al (2021)	US	indoor vaping restriction; taxation; Tobacco 21	state	DID	students (grade 9-12)	ever and current e-cigarette use	YRBSS	8
Chung-Hall et al (2020)	seven European countries	TPD	country	pre-post	adult smokers	policy support	ITC 6 European Country Survey and ITC Four Country Smoking and Vaping Survey	7
Colditz et al (2021)	US/Pennsylvania	taxation	state	ITS	retailers	the number of open vape shops	NA	6
Cotti et al (2022)	US	taxation	state	IV	NA	e-cigarette sales	NRSD	9
Dai and Hao (2022)	US	flavor ban	country	ITS	NA	relative search volume of JUUL and Puff Bars	Google Trends data	8
Dai et al (2021)	US/Kansas	Tobacco 21	locality	DID	students (grade 6,8,10,12)	e-cigarette use in last month	Kansas Communities That Care Student Survey	7
Driller et al (2021)	US	packaging	state	ITS	NA	the number of nicotine toxicity reported to the CPCS.	suspected nicotine toxicity cases reported to the California Poison Control System	7
Ferrell et al (2020)	US/Florida	age restriction	state	DID	students (grade 6-12)	e-cigarette use in last month and perceptions	Florida Youth Tobacco Survey	8
Friedman et al (2021)	US	indoor vaping restriction	locality	DID	adults (18-54 age)	e-cigarette use in last month	NHIS	8
G. Martin et al (2021)	Canada	advertising restrictions	province	pre-post	retailers	the number and density of vaping advertisements surrounding secondary schools	NA	6

García-Ramírez et al (2022)	US/California	Tobacco 21	state	pre-post	students (grade 7,9,11)	e-cigarette use in last month	California Healthy Kids Survey	8
Girvalaki et al (2020)	nine European member states	TPD	country	pre-post	NA	labelling, packaging and technical design characteristics of e-liquid	NA	6
Grube et al (2021)	US/California	Tobacco 21	state	ITS	students (grade 7,9,11)	e-cigarette use in last month	California Healthy Kids Survey	7
Hammond et al (2020)	Canada	advertising restrictions	province	DID	adolescents and young adults (16-19)	e-cigarette marketing exposure, e-cig use in last month	ITC Youth Tobacco and Vaping Survey	8
Hammond et al (2022)	US	flavor ban	country	DID	youth vapers (16-19 years)	e-cigarette flavor, device, and brand used most often by current vapers	ITC Youth Tobacco and Vaping Survey	7
Han et al (2021)	US	taxation	state	DID	young adults (18-24)	e-cigarette use (daily, some days)	the Tobacco Use Supplement to the Current Population Survey	9
Hawkins et al (2021)	US/Massachusetts	flavor ban; indoor vaping restriction; Tobacco 21	locality	DID	students (grade 9-12)	e-cigarette use in last month	the Massachusetts Youth Health Survey	9
Holmes et al (2022)	US/California	flavor ban	locality	pre-post	retailers	availability and advertising of flavored tobacco products	another study (Holmes, L.M.)	8
Kahnert et al (2020)	six European countries	TPD	country	pre-post	adult smokers	self-exposure to advertising and promotions	ITC Six European Country Survey	7
Katchmar et al (2021)	US/Massachusetts	taxation; comprehensive ban	state	ITS	NA	e-cigarette weekly sales	Nielsen ScanTrack data	9
Kephart et al (2020)	US/Massachusetts	flavor ban	locality	pre-post	retailers	Flavored tobacco product availability and advertisements	MTCP	6
Kingsley et al (2019)	US/Massachusetts	flavor ban	locality	DID	students (grade 9-12)	e-cigarette use in last month	MTCP	7
Kingsley et al (2020)	US/Massachusetts	flavor ban	locality	DID	retailers	flavored product availability	MTCP	8
Kingsley et al (2021)	US/Massachusetts	flavor ban	locality	DID	students (grade 9-12)	e-cigarette use in last month	MTCP	8

Kowitt et al (2022)	Indonesia	taxation	country	pre-post	adult smokers	e-cigarette use (weekly)	NA	6
Laestadius et al (2020)	US	advertising restrictions	country	ITS	NA	warning statement of posts	NA	8
Lal et al (2021)	India	comprehensive ban	country	pre-post	NA	e-cigarette availability in websites	NA	5
Lee et al (2020)	UK	TPD	country	pre-post	adult smokers	current use and awareness of regulations	a longitudinal online survey of smokers, ex-smokers, and vapers from the United Kingdom.	5
Lu et al (2022)	US	flavor ban	country	pre-post	NA	Twitter users perception of policy and e-cigarettes	Twitter	7
Moore et al (2020)	Wales, England and Scotland	TPD	constituent country	ITS	students (grade 9,11)	e-cigarette use (weekly)	School Health Research Network/Health Behaviour in School-aged Children surveys in Wales	8
Mourik et al (2019)	Netherlands	TPD	country	DID	smokers >=15 years	noticed the warnings and leaflets in last month and perceptions towards e-cigarette	ITC Netherlands Survey	7
Nguyen (2020)	Canada	age restriction	province	DID and DDD	youth (15-25 years) and students (6-12 grades)	use in last month, difficulty of access to e-cigarettes, harm perception, and use of social sources of e-cigarettes.	the Canadian Tobacco, Alcohol and Drugs Survey (CTADS) and the Canadian Student Tobacco, Alcohol and Drugs Survey (CSTADS)	9
Nguyen and Bornstein (2021)	Canada	indoor vaping restriction	province	DID	adults (age >19)	e-cigarette use in last month	Canadian Tobacco Use Monitoring Survey (CTUMS) and Canadian Tobacco, Alcohol and Drugs Survey (CTADS).	7
Nikitara et al (2020)	six European countries	TPD	country	pre-post	adult smokers	e-cigarettes (weekly); Noticing and reading e-cigarette health warnings and leaflets	ITC Six European Country Survey	6
Olson et al (2022)	US/Minnesota	flavor ban	locality	pre-post	students (grade 6-12)	e-cigarette use in last month	the Minnesota Youth Tobacco Survey and the Minnesota Student Survey	8

Pesko et al (2020)	US	taxation	locality	DID	adults	e-cigarette use (use daily or some days)	BRFSS and NHIS	8
Polanska and Kaleta (2021)	Poland	advertising restrictions	country	pre-post	retailers	the presence of ads and minimum age sign	NA	5
Roberts et al (2022)	US/Ohio	Tobacco 21	state	pre-post	first-year students aged >=18	e-cigarette use in last month	a prospective cohort study that examined tobacco use among undergraduate students in Columbus, Ohio.	6
Ruokolainen et al (2022)	Finland	TPD	country	pre-post	adolescents and adults (15-69 years)	e-cigarette use (Daily or almost daily use and occasional use were classified as current use)	population-based drug surveys, conducted every fourth year in Finland	6
Schiff et al (2021)	US/California	Tobacco 21	state	pre-post	young adults (18-19)	e-cigarette use in last month and purchase locations	the Southern California Children's Health Study	6
Sun et al (2022)	US/New York	flavor ban	state	pre-post	NA	the attitudes towards policy	Twitter	9
Taylor et al (2022)	England	TPD	country	pre-post	adult smokers	noticing warnings and leaflets, recall of warnings about nicotine and concerns about using NVP due to noticing warnings	ITC Four Country Smoking and Vaping Surveys	8
Wilhelm et al (2022)	US/Minnesota	Tobacco 21	locality	DID	students (grade 8,9,11)	e-cigarette use in last month	Minnesota Student Survey	7
Yang et al (2022)	US	indoor vaping restriction	state	DID	adults	e-cigarette use in last month	Population Assessment of Tobacco and Health study	9

NRSD: the NielsenIQ Retail Scanner Dataset; NHIS: the National Health Interview Survey; ITC: International Tobacco Control (ITC) Policy Evaluation Project; YRBSS: Youth Risk Behavior Surveillance System; MTCP: The Massachusetts Tobacco Cessation and Prevention programme DID: difference-in-difference analysis; DDD: triple-difference analysis; ITS: interrupted time series analysis; IV: instrumental variable; NA: not applicable; TPD: Tobacco Product Directive.

Table S5. Main findings of included studies by policy classification

Study ID	Study design	Comparator	Main findings
**Flavor restrictions**			
Ali et al (2022)	DID	states with the restrictions compared with 35 states without these restrictions; pre-post	Statewide restrictions on non-tobacco-flavored e-cigarette sales were associated with the following reductions in mean 4-week total e-cigarette sales in intervention states compared with control states from October 2019 to December 2020: 30.65% (95% CI, 24.08%-36.66%) in New York, 31.26% (95% CI, 11.94%-46.34%) in Rhode Island, and 25.01% (95% CI, 18.43%-31.05%) in Washington.<br>The increases in sales of tobacco-flavored e-cigarettes were approximately 40.52%, 43.08%, and 49.17%of the observed total sales decreases in Washington, New York, and Rhode Island, respectively.
Dai and Hao (2022)	ITS	time trend	the RSV for JUUL dropped sharply (rate of change=−8.8 per week, 95%CI=-12.9 to -4.7; p=0.01) from 11 September 2019 when Trump administration announced plans to ban flavors to 17 October 2019 when JUUL Labs announced to halt online sales of some flavored products, and the RSV resumed the decreasing trend after FDA announced enforcement policy of cartridge-based e-cigarettes on 2 January 2020(rate=-2.1, -3 to -1.2,p<0.01). In comparison, the RSV for Puff Bar started to increase after 11 September 2019 with a low rate of change (0.6) until 17 October 2019. After that, the increase in RSV for Puff Bar accelerated (p=1.6, 17 Oct 2019–02 Jan 2020; p=3.0,02 Jan 2020–22 Feb 2020)
Hammond et al (2022)	DID	US versus Canada and England; pre-post	Disposable e-cigarettes (exempt from flavor restrictions) increased to a greater extent among vapers in the United States (13.2% to 36.8%) versus Canada (7.7% to 14.2%; AOR = 2.01; 95% CI = 1.33, 3.04) and England (10.8% to 16.4%; AOR = 2.33; 95% CI = 1.52, 3.57).<br>no significant differences were observed in the proportion of youth vapers who usually used restricted/unrestricted flavors before (2017–2019) or after (August 2020) restrictions were implemented in US, same as vapers in Canada and England.
Hawkins et al (2021)	DID	students exposed to the law compared with whom not; pre-post	Flavored tobacco product restrictions also were associated with a reduction in adolescent e-cigarette use (Coefficient:-0.87; 95% CI: -1.68 to -0.06).
Holmes et al (2022)	pre-post	retailers in cities with a flavor policy compared with them not; pre-post	retailers in cities enacted a ban: significant reduction in availability of vape pens (19.2% to 9.4%) and Blu menthol e-cig (53.3% to 6.2%), while E-cigarette not(58.3% to 55.8%); the availability of exterior e-cigarette ads (32.0% to 9.7%) and interior e-cigarette ads(39.6% to 19.3%) decreased<br>control group: significant increase in availability of e-cigarettes(76.5 to 77.6%), while it of Blu menthol e-cig(64.3 to 34.3%) and exterior e-cigarette ads(41.7 to 20.4%) decreased
Kephart et al (2020)	pre-post	pre-post	Total number of flavored vaping products inventoried decreased from 1135 in baseline to 17 in follow-up.
Kingsley et al (2019)	DID	students in the control municipality without the policy; pre-post	Compared with Malden, the regulation in Lowell resulted in a insignificant decrease in current flavored e-cigarette use (-3.1%, 95%CI=-6.9% to 0.7%) and non-flavored e-cigarette use (-1.1%, 95%CI=-4.5% to 2.3%).
Kingsley et al (2020)	DID	cigarette availability; pro-post	From pre-policy to post-policy implementation period, in both wave 1 and wave2, flavored vaping product availability significantly decreased (22,5%, p=0.03; 52.9%, p<0.01, respectively)
Kingsley et al (2021)	DID	Attleboro (earlier adoption municipality; EA) and Salem (later adoption municipality; LA) compared with Gloucester(without a FTR); pre-post	compared with Gloucester, in Attleboro, insignificant decrease was found in ever and current non-flavored/menthol e-cigarette use(ever: -4.6%, -10.3% to 1.1%; current: -4.0%, -8.2% to 0.1%), and current flavored use significantly decreased (-7.6%, -12.3%-2.8%) In Salem, significant decreases were found in ever e-cigarette use(both flavored (-12.5%, -20.5% to-4.5%))and non-flavored(-12.3%, -19.7% to -5.0%))) and current non-flavored/menthol e-cigarette use(-8.3%, -13.7% to -2.9%) and flavored use (-11.6%, -17.7% to -5.4%)

Lu et al (2022)	pre-post	pre-during-post	The proportion of negative sentiment tweets about e-cigarettes significantly increased after the announcement of the FDA flavor enforcement policy compared with before the announcement of the policy(before: 27.5% (95% CI 27.4%-27.6%), during: 39.4% (95% CI 39.2%-39.5%), after: 41.5% (95% CI 41.4%-41.6%)), and the proportion of tweets with positive sentiment toward e-cigarettes decreased significantly (P<.001). In contrast, the overall sentiment toward the FDA flavor enforcement policy became less negative, from 22.8% (95% CI=22.3%-23.2%) to 24.5% (95% CI=24.0%-25.0%) and 26.2% (95% CI=25.6%-26.7%).
Olson et al (2022)	pre-post	Twin Cities area (including Minneapolis and St. Paul) versus the rest of the state of Minnesota (ROS); pre-post	between 2014 and 2017, the prevalence of e-cigarette use increased in the Twin Cities from 11.1% to 14.9% (+34.1%, p < .05), while in ROS from4.8% to 10.2(+114.0%) (p < .05).between 2016 and 2019, E-cigarette use increased in both geographies, from 10.5% to 15.7% (+49.5%) in the Twin Cities and from 10.0% to 18.8% (+88.9%) in ROS.
Sun et al (2022)	pre-post	tweets from New York compared with tweets from other states	after the announcement of the New York State flavor policy, in both New York State and other states, the proportion of negative tweets on e-cigarettes increased from 34.07% (4531/13,299) to 44.58% (18,451/41,390) and from 32.48% (14,320/44,090) to 44.40% (64,262/144,734), respectively, while positive tweets decreased significantly from 39.03% (5191/13,299) to 32.86% (13,601/41,390) and from 42.78% (18,863/44,090) to 33.93% (49,105/144,734), respectively. The majority of tweets related to the New York State flavor policy were negative both before and after the announcement of this policy in both New York (87/98, 89% and 3810/4565, 83.46%, respectively) and other states (200/255, 78.4% and 12,695/15,569, 81.54%, respectively)
**Vape-free policies**			
Choi et al (2021)	DID	students in states having ESF policies, any e-cigarette excise tax and T21 compare with not; pre-post	current use: decreased in states with ESF policies during 2017-2019 (-45.4%, AOR=0.53, 0.41 to 0.68), increased in states without ESF policies (68.1%, AOR=2.60, 2.37 to 2.87), time*policy interaction p<0.01.
Friedman et al (2021)	DID	adults living in counties with vape-free policies compared with whom not; pre-post	Adding vaping restrictions to smoke-free worksite laws did not yield further reductions in recent vaping (β=0.008,95%CI: -0.021to 0.036, P = 0.568) and counteracted over half of the estimated association with current smoking relative to smoke-free policies alone (β = 0.030, 95% CI: -0.028 to 0.088, P = 0.301).<br>Similar as vape-free restaurant laws.
Hawkins et al (2021)	DID	students exposed to the law compared with whom not; pre-post	There were no significant associations between vape-free laws(coefficient=0.23 ,-0.80 to 1.25) with e-cigarette use.
Nguyen and Bornstein (2021)	DID	adult exposed to the ban compared with whom not; pre-post	After the bans, e-cigarette use in the past 30 days did not change significantly in provinces with a ban compared with provinces without a ban (0.004; 95% CI -0.025 to 0.032; p=0.783), as well as experimental use
Yang et al (2022)	DID	participants living in states with aerosol-free policies compared with participants living in states without policies; pre-post	Across all regression models, there were no significant associations between the inclusion of e-cigarettes in comprehensive smoke-free indoor air laws and e-cigarette use behaviors (ps ranged from 0.301 to 0.831).
**Taxation**			
Amato and Boyle (2016)	ITS	The Minneapolis compared with The Saint Louis (control)	Minneapolis prices of the 4 most popular products increased a mean of 21.44% after the tax increase.<br>Total sales in the 2 time periods immediately following taxes were significantly greater than expected in Minneapolis(P<0.01), before dropping significantly below expected values for the 4 final time periods. Sales increases were driven by gains for Blu e-cigarettes, then owned by Lorillard, compared to sales decreases for NJOY products.

Choi et al (2021)	DID	students in states having ESF policies, any e-cigarette excise tax and T21 compare with not; pre-post	current use: increased in both states with excise taxes (86.4%, AOR=2.94, 2.45 to 3.52) and states without (44.5%, AOR=2.07, 1.84 to 2.32)
Colditz et al (2021)	ITS	pre-post	The number of listed vape shops increased in a linear fashion by a magnitude of 23% in less than 2 years, roughly a quarter (22%-29%) of vape shops to be noncompliant with maintaining a valid ENDS retail license
Cotti et al (2022)	IV	localities adopting taxes compared with them not; pre-post	A $1.00 increase in e-cigarette taxes raises e-cigarette prices by $0.90(SE0.08).<br>an e-cigarette own-price elasticity of demand was -2.2, and approximately twice as elastic of demand for non-mentholated flavored e-cigarettes compared to non-flavored and mentholated e-cigarettes.<br>a $1.00 increase in e-cigarette taxes reduces e-cigarette sales by 919 ml(SE285) per 100,000 state adult residents.
Han et al (2021)	DID	adults living in localities with excise taxes compared with whom not; pre-post	Respondents living in states with excise tax policy showed significantly lower increase in use prevalence during the study period (interaction between within-state changes and between-state differences: adjusted OR (AOR)=0.53, 95% CI=0.28 to 0.99).
Katchmar et al (2021)	ITS	weekly sales in the Greater Boston area compared to it in the USA; pre-post	no significant changes in the level or trend of year-on-year e-cigarette sales per capita in the Greater Boston convenience market after House Bill No. 4196 implementation(p value for level change=0.896, p value for trend change=0.192). Post-trend analysis showed the rate of decline in sales in the Greater Boston area was significantly lower than that of the USA (diff=1.19, P=0.0078).
Kowitt et al (2022)	pre-post	pre-post	Following the e-liquid tax, participants reported paying a 4.4% higher price for e-liquid (p=0.02), and an average 0.5-day decrease in the number of days they used e-cigarettes in the past week (from 5.7 to 5.2, p<0.001), and the proportion of daily e-cigarette users decreased (75.9% to 63.6%; p<0.001).
Pesko et al (2020)	DID	adults living in localities with excise taxes compared with whom not; pre-post	A $1.00 increase in tax per fluid ml of vaping liquid reduces the probability of current vaping by 0.52 ppts (95%CI=-0.011, -0.0006p<0.10) or 15.3% and the probability of daily vaping by 0.17 ppts (p>0.10) or 14.2%; increases daily smoking propensity by 0.6 ppts (p<0.05) or 5.3%.<br>It is potentially driven by young adults. Among them, a $1.00 increase in the standardized e-cigarette tax increases daily smoking by 1.2 ppts (p<0.01) or 8.8%, reduces current vaping by 1.8 ppts (p<0.01), or 26.0%, and reduces daily vaping by 0.6 ppts (p<0.01), or 24.8%.
**Comprehensive ban**			
Ali et al (2022)	DID	states with the restrictions compared with 35 states without these restrictions; pre-post	In Massachusetts, prohibition of all e-cigarettes reduced 94.38% (95% CI, 93.37%-95.23%) sales from November 3 to December 1, 2019, compared with the control states.
Katchmar et al (2021)	ITS	weekly sales in the Greater Boston area compared to it in the USA; pre-post	The Greater Boston area saw a significant decrease in the level of sales as compared to the USA (β=-14.2, p value for level change=0.029; β=-1.2, p value for trend change=0.208) after Massachusetts ban on e-cigarettes, while there was a significant increase in the trend of e-cigarette sales after ban lifting (β=2.8, p value for trend change=0.000)
Lal et al (2021)	pre-post	pre-post	All inspected website, whether comprehensive e-commerce portals or dedicated ENDS marketing platforms still sold vaping products and accessories. E-cigarette portals still had no age restrictions.
**Age restrictions**			

Ferrell et al (2020)	DID	adult students; pre-post	Compared to the data from spring of 2014, the minimum-age policy enacted on July 1, 2014 did not lead to a significant decrease in Floridas high school and middle school students ever ENDS use (14.9% in 2014 vs 25.8% in 2015) and curr ent ENDS use (7.5% in 2014 vs 12.4% in 2015), the interaction term were not significant.<br>More students were confident about ENDS lower harm (32.4% vs 28%) and greater harm (11.0% vs 7.7%) than cigarette, were not as addictive as cocaine or heroin (21.8% vs 12.0%) and that this tobacco product was easy to quit (34.5% vs 29.6%), ENDS users had fewer friends (34.3% vs 28.7%); ENDS use did not help people feel more comfortable at social situations (37.0% vs 32.6%); and ENDS did not relieve stress (32.0% vs 26.1%)
Nguyen (2020)	DID and DDD	students in provinces with the ban compared with whom not; targeted age group compared with untargeted age group; pre-post	DDD: the ban resulted in 3.1 percentage points (95% CI, 0.2-6.0; P = .04) reduction, or 79% in e-cig use.<br>DD: Youths in provinces with a ban were 2.6 percentage points (95% CI, 1.5-3.7; P = .001), or 18%, less likely to believe that regular e-cigarette use poses no harm and 6.2 percentage points (95% CI, 1.1-11.4; P = .02), or 16%, more likely to self-report greater difficulty in obtaining e-cigarettes. Among youths who reported using e-cigarettes, the likelihood of obtaining e-cigarettes from social sources was 17.3 percentage points (95% CI, 5.2 -29.4; P = .01), or 29%, higher in provinces with a ban.
**Tobacco 21**			
Choi et al (2021)	DID	students in states having ESF policies, any e-cigarette excise tax and T21 compare with not; pre-post	current use: states with T21 policies showed non-significant changes (AOR=0.83, 0.62 to 1.15); states without T21 policies increased (52.6%, AOR=2.26, 2.05 to 2.49), interation p<0.01
Dai et al (2021)	DID	students exposed to the law compared with whom not; pre-post	prevalence of current use increased from 8.2% in 2018 to 12.6% in 2019. The increase was larger in rural areas (from 6.7% in 2018 to 13.4% in 2019) than in urban areas (9.8%–11.9%), with a significant interaction effect of year × urbanicity/T21 group (P < .0001).<br>In urban areas, e-cigarette use increased significantly for middle school students in T21 areas (3.3%–4.5%; aOR=1.4, 1.1 to 1.9, P = .01) and all students in non-T21 areas (8.1%–12.0%; aOR=1.8, 1.6 to 2.0, P < .0001). In rural areas, the increase was smaller in T21 areas (7.9%–10.8%, difference = 3.0%) than in non-T21 areas (6.5%–13.7%, difference = 7.1%).
García-Ramírez et al (2022)	pre-post	non-SM adolescents; pre-post	T21 was associated with increases in past 30-day use (OR=1.06, 95%CI=1.03-1.08) and decrease in lifetime use (OR=0.63, 95%CI=0.62-0.64)<br>T21 was associated with significant reductions in lifetime e-cigarette use (SM youth: OR = 0.66, 0.64-0.68, non-SM: OR=0.64, 0.61-0.64). For 30-day e-cigarette , SM youth showed no significant increases, whereas non-SM youth showed significant increases (OR = 1.06, 1.04-1.09) following T21
Grube et al (2021)	ITS	pre-post	Multilevel mixed effects logistic regression analyses showed that T21 was associated with reduced prevalence of lifetime e-cig use (OR=0.72, 95%CI=0.71-0.73) and increases in prevalence of past month e-cigarette use in the overall student population (OR=1.09, 95%CI= 1.07-1.11).<br>Moderation analyses indicated differences by racial and ethnic groups. T21 was associated with reduced prevalence of lifetime e-cig use (OR=0.85, 95%CI=0.83-0.87) and increases in prevalence of past month e-cigarette use in Non-Latinx white (OR=1.43, 95%CI= 1.39-1.47)
Hawkins et al (2021)	DID	students exposed to the law compared with whom not; pre-post	There were no significant associations between tobacco 21 policies(coefficient=0.22, -0.66 to 1.10)with e-cigarette use.
Roberts et al (2022)	pre-post	pre-post	e-cigarette past 30-day use rapidly increased during the same period. (from 7.4% to 34.5%)

Schiff et al (2021)	pre-post	per-post	prevalence of current e-cigarette use decreased after T21 (from 12.9% to 9.4%) while ever use increased (from 12.9% to 42.1%)<br>current users reporting that they purchased their e-cigarettes from a vape shop decreased (pre-T21: 37.8%; post-T21:29.1%), whereas the proportion reporting not purchasing products increased (pre-T21: 51.8%; post-T21: 57.5%), though these were insignificant
Wilhelm et al (2022)	DID	student in localities with a T21 policy compared with whom not; pre-post	After controlling prevalence in 2016, T21-exposed eighth and ninth-grade students in2019 had significantly lower odds of e-cigarettes use (aOR = 0.78, 95% CI: 0.71, 0.85).T21-exposed eleventh-grade students did not differ significantly in it relative to their unexposed peers.
**TPD**			
Barhdadi et al (2021)	pre-post	pre-during-post	Although not all manufacturers managed to produce and label their products accurately, nicotine labeling discrepancies have decreased over time. Moreover, also the number of e-liquids, containing high-risk VOCs (10% in 2016 vs. none of the samples in 2017-2018), caffeine (16% in 2017 vs. 5% in 2018), and diacetyl and acetylpropionyl (50% in 2017 vs. 27% in 2018 of sweet-flavored samples) diminished over time
Chung-Hall et al (2020)	pre-post	pre-post	Banning EC use in smoke-free places was supported by 53.1% in 2016 and 54.6% in 2018 with a significant increase in Greece (51.7–66.0%) and a decrease in Spain (60.1–48.6%). Restricting EC/e-liquid nicotine content was supported by 52.2 and 47.4% in 2016 and 2018, respectively, with a significant decrease in England (54.2–46.5%) and Romania (52.5–41.0%). An EC promotion ban was supported by 41.1 and 40.2%, with no significant change in support in all the countries. A flavor ban was supported by 33.3% and 32.3% with a significant increase in Hungary (34.3–43.3%).
Girvalaki et al (2020)	pre-post	pre-post	The compliance with TPD increased: text-only warnings increased (32.7% pre vs 86.0% post, $p<0.001$), child-resistant fastenings (93.3% vs 100.0% , $p=0.016$), tamper-proof vials (58.9% vs 86.9%, $p<0.001$) and maximum refill volume ≤10 mL in vials (86.9% vs 94.4%, $p=0.008$); the inclusion of a leaflet (26.2% vs 53.3%, $p<0.001$), refilling instructions (28.0% vs 51.4%, $p<0.001$) and health warnings on the box, vial or leaflet (32.7% vs 86.0%, $p<0.001$). 86.0% of products had a warning label in the post-TPD phase in comparison to 32.7% of products before the implementation of the TPD ($p<0.001$).
Kahnert et al (2020)	pre-post	pre-post	The percentage of respondents who reported having been exposed to ECAPS increased in four of the six countries from W1 to W2, while a decrease was seen in Poland and Romania.<br>adjusted GEE models shows: In the pooled sample, overall exposure to ECAPS in any medium or locality was significantly higher at W2 than at W1 (aOR = 1.25, 95% CI: 1.09–1.44), with great variation in change of exposure to ECAPS between waves across countries.
Lee et al (2020)	pre-post	pre-post	Wave 5 had a statistically greater proportion of current vapers compared to wave 4(from 29.9% to 32.1%, P=0.03)<br>Higher proportions used TPD-compliant refill volumes (60.0%-73.7%, $\chi2(1) = 10.9$, $p = .001$) and nicotine concentrations (89.2%-93.9%, $\chi2(1) = 7.41$, $p = .007$) in wave 5 than wave 4, with little change for tank or cartridge volumes (77.1-75.5%, $\chi2(1) = 0.38$, $p = .540$).<br>No significant differences were seen in the proportion using TPD-compliant volumes for cartridges or tanks in their e-cigarette devices before and after TPD implementation.

Moore et al (2020)	ITS	pre-post	Regular e-cigarette use increased from 2015 to 2017 in Wales ((OR: 1.07; 95% CIs: 0.98-1.17)).<br>In Wales, growth in ever use did continue post-TPD after adjusting for ever smoking (trend: OR=0.93; 95%CI=0.88 to 0.98).
Mourik et al (2019)	DID	vapers compared with tobacco-only smokers; pre-post	compared to tobacco-only smokers, a higher proportion of e-cigarette users noticed the warning (OR = 4.006, p < 0.001) and knew the leaflet (OR = 5.530, p < 0.001).<br>respondents reported higher scores on perceptions regarding the addictiveness (from 2.70 to 2.88, β=0.141 (0.088 to 0.194)) and toxicity(from 2.49 to 2.48 β=0.069 (0.018 to 0.120)) of e-cigarettes after implementing the new legislation than before. E-cigarette users showed a greater increase in scores on the perception regarding the addictiveness of e-cigarettes (β = 0.457, p = 0.045) than tobacco-only smokers (β = 0.135, p < 0.001). Also, e-cigarette users showed no change in scores on the perception regarding the toxicity of e-cigarettes
Nikitara et al (2020)	pre-post	pre-post	there was a significant increase in respondents reporting noticing and reading health and product safety information on leaflets inside e-cigarette packaging (8.39–11.62%, P < 0.001), but no significant changes between waves of respondents reporting noticing or reading warning labels on e-cigarette packages/vials. Among those who noticed leaflets in the packaging, there was a significant increase in those who reported reading the information contained in the leaflets (2.8–3.9%, P < 0.05). Percentage of respondents reporting the ability to adjust the settings on their vaping device increased slightly (1.2–1.8%, P < 0.05).<br>there was no significant change in the proportion of respondents who reported current (daily or weekly) e-cigarette use (from 1.66% to 2.17%, p=0.105), nor in those who reported use of flavored e-liquids (including tobacco flavor), using nicotine-containing e-liquids, or ever mixing e-liquids (proportion increased, P>0.05).
Ruokolainen et al (2022)	pre-post	pre-post	no significant associations between current use and survey year (OR=0.98, 0.67-1.43), the odds of experimental use and former use were higher in 2018 than in 2014, (OR=1.28, 1.06-1.54; OR= 2.75, 1.77-4.25, respectively)
Taylor et al (2022)	pre-post	smokers in England compared with smokers in control countries (Canada, the US and Australia); pre-post	Noticing warnings increased in England from 4.9% (2016) to 9.4% (2018) (adjusted OR/AOR=1.64, 95% CI=1.15-2.36); larger than changes in Canada (AOR=2.51, 95% CI=1.71-3.69) and the US (AOR=2.22, 95% CI=1.45-3.39). Recall of a nicotine warning increased in England from 86% to 94.9% (AOR=5.50, 95% CI=1.57-19.27) but not significantly elsewhere. Noticing leaflets increased in England from 14.6% to 19.1% (AOR=1.42, 95% CI=1.15-1.74); larger than in Canada (AOR=1.42, 95% CI=1.12-1.79), the US (AOR=1.55, 95% CI=1.17-2.06) and Australia (AOR=1.51, 95% CI=1.02-2.22). Among those noticing warnings, concern about NVP use did not change significantly between 2016 and 2018 (all countries p>0.081)
**Advertising restrictions**			
G. Martin et al (2021)	pre-post	pre-post	Prior to the ban, there were 266 vaping advertisements within 800 m of secondary schools. After the ban, this was reduced to 58, a 78.2% reduction. The mean number of vaping advertisements surrounding schools significantly decreased from 18.1 before the ban to 3.6 after the ban (p < 0.001)

Hammond et al (2020)	DID	participants living in provinces with differing strengths of marketing restrictions; pre-post	The likelihood of often noticing promotions increased from 2017 to 2019 for all provinces except those with high restrictions (high: AOR=1.27, 95% CI=0.95–1.71, P = .10). increase in often noticing promotions between 2017 and 2019 was substantially greater among provinces with fewer restrictions compared with provinces with the most restrictions (moderate-high: AOR = 2.00, 95% CI = 1.25–3.22, P =.004; moderate: AOR = 2.66, 95% CI =1.22–5.80, P = .01; low-moderate: AOR = 1.88, 95% CI = 1.33–2.65, P<.001; low restrictions: AOR = 2.93,95% CI = 1.89–4.54, P<.001).<br>respondents in provinces with no restrictions on specific channels were more likely to notice promotions in these places, and this difference was larger in 2019 than 2017.<br>the prevalence of current vaping increased from 2017 to 2019 for all provinces (high restrictions: AOR =2.09, 95% CI = 1.52–2.87, P , .001; moderate-high: AOR = 2.25, 95% CI =1.45–3.50, P , .001; moderate: AOR =2.70, 95% CI = 1.24–5.86, P = .01;low-moderate: AOR = 2.84, 95% CI =2.26–3.57, P , .001; low: AOR = 2.28,95% CI = 1.59–3.25, P , .001).
Laestadius et al (2020)	ITS	pre-post	FDA compliant warnings increased from 0% to 13.6% of overall promotional posts after August 2018. Among US-based posts, 36.4% used the warnings. The share of posts made by US Instagram users decreased from38% t0 27.9%. Promotional strategies and products did not significantly change.
Polanska and Kaleta (2021)	pre-post	pre-post	A significant increase in the presence of any e-cigarette ads in e-cigarette retailers, including e-cigarette displays, illuminated banners and video screens, was observed in 2019 as compared to 2014 (90% vs. 30%; 89% vs. 20%; 31% vs. 2%; 31% vs. 0.5%; p < 0.001).<br>The minimum age signs for e-cigarettes were not found in 2014 (as this was not regulated by law at that time) and in 29% of POS checked in 2019<br>more e-cigarettes were placed in close proximity to products for children (16% in 2014 vs. 27% in 2019; p < 0.001)
**Packaging**			
Driller et al (2021)	ITS	pre-post	Implementation of the CNPPA policy was not significantly associated with reduced exposures, monthly exposures increased from 18.2 to 21.2. Exposures for e-cigarettes increased significantly after the 2017 Food and Drug Administration Compliance Policy (p=0.003, coefficient =0.61), monthly exposures increased from 18.4 to24.0.
**Retail licensing policy**			
Azagba et al (2019)	DID	students in Pennsylvania compared with students in control states (New York and Virginia); pre-post	Compared with New York adolescents, e-cigarette use among Pennsylvania adolescents reduced by 5.2 percentage points(SE=0.020) in 2017, and a corresponding 21.6% decrease from its baseline prevalence level in 2015.<br>Compared with Virginia, reduced by 7.4 percentage points (SE=0.018) decrease in e-cigarette use in Pennsylvania when compared with Virginia (SE=0.018; 30.7% relative decrease from the baseline prevalence)

Table S6. Quality assessment based on the JBI Critical Appraisal checklist for quasi-experimental studies

Study ID	Q1	Q2	Q3	Q4	Q5	Q6	Q7	Q8	Q9	Total score
Ali et al (2022)	Yes	Yes	No	Yes	Yes	NA	Yes	Yes	Yes	9
Amato and Boyle (2016)	Yes	Yes	No	Yes	Yes	NA	Yes	Yes	Yes	9
Azagba et al (2019)	Yes	Yes	Yes	Yes	No	Yes	Yes	Yes	Yes	7
Barhdadi et al (2021)	No	Yes	No	No	Yes	NA	Yes	Yes	Yes	7
Choi et al (2021)	Yes	Yes	No	Yes	No	Yes	Yes	Yes	Yes	8
Chung-Hall et al (2020)	Yes	Yes	No	No	No	Yes	Yes	Yes	Yes	7
Colditz et al (2021)	Yes	Yes	Yes	No	No	NA	Yes	Yes	Yes	6
Cotti et al (2022)	Yes	Yes	No	Yes	Yes	NA	Yes	Yes	Yes	9
Dai and Hao (2022)	Yes	Yes	No	No	Yes	NA	Yes	Yes	Yes	8
Dai et al (2021)	Yes	Yes	Unclear	Yes	No	Yes	Yes	Yes	Yes	7
Driller et al (2021)	Yes	Yes	No	No	Yes	NA	Yes	Unclear	Yes	7
Ferrell et al (2020)	Yes	Yes	No	Yes	No	Yes	Yes	Yes	Yes	8
Friedman et al (2021)	Yes	Yes	No	Yes	Yes	No	Yes	Yes	Yes	8
G. Martin et al (2021)	No	Yes	No	No	No	Yes	Yes	Yes	Yes	6
García-Ramírez et al (2022)	Yes	Yes	Yes	Yes	Yes	Yes	Yes	Yes	Yes	8
Girvalaki et al (2020)	No	Yes	No	No	No	NA	Yes	Yes	Yes	6
Grube et al (2021)	Yes	Yes	Yes	No	Yes	Yes	Yes	Yes	Yes	7
Hammond et al (2020)	Yes	Yes	No	Yes	No	Yes	Yes	Yes	Yes	8
Hammond et al (2022)	Yes	Yes	Yes	Yes	No	Yes	Yes	Yes	Yes	7
Han et al (2021)	Yes	Yes	No	Yes	Yes	Yes	Yes	Yes	Yes	9
Hawkins et al (2021)	Yes	Yes	No	Yes	Yes	Yes	Yes	Yes	Yes	9
Holmes et al (2022)	Yes	Yes	No	Yes	No	Yes	Yes	Yes	Yes	8
Kahnert et al (2020)	Yes	Yes	No	No	No	Yes	Yes	Yes	Yes	7

Katchmar et al (2021)	Yes	Yes	No	Yes	Yes	NA	Yes	Yes	Yes	9
Kephart et al (2020)	No	Yes	No	No	No	Yes	Yes	Yes	Yes	6
Kingsley et al (2019)	Yes	Yes	No	Yes	No	No	Yes	Yes	Yes	7
Kingsley et al (2020)	Yes	Yes	No	Yes	No	Yes	Yes	Yes	Yes	8
Kingsley et al (2021)	Yes	Yes	No	Yes	No	Yes	Yes	Yes	Yes	8
Kowitt et al (2022)	No	Yes	No	No	No	Yes	Yes	Yes	Yes	6
Laestadius et al (2020)	Yes	Yes	No	No	Yes	NA	Yes	Yes	Yes	8
Lal et al (2021)	No	Yes	No	No	No	Yes	Yes	Yes	No	5
Lee et al (2020)	No	Yes	No	No	No	No	Yes	Yes	Yes	5
Lu et al (2022)	No	Yes	No	No	Yes	NA	Yes	Yes	Yes	7
Moore et al (2020)	Yes	Yes	No	No	Yes	Yes	Yes	Yes	Yes	8
Mourik et al (2019)	Yes	Yes	No	No	No	Yes	Yes	Yes	Yes	7
Nguyen (2020)	Yes	Yes	No	Yes	Yes	Yes	Yes	Yes	Yes	9
Nguyen and Bornstein (2021)	Yes	Yes	Unclear	Yes	Yes	No	Yes	Yes	Yes	7
Nikitara et al (2020)	No	Yes	No	No	No	Yes	Yes	Yes	Yes	6
Olson et al (2022)	Yes	Yes	No	Yes	No	Yes	Yes	Yes	Yes	8
Pesko et al (2020)	Yes	Yes	No	Yes	Yes	No	Yes	Yes	Yes	8
Polanska and Kaleta (2021)	No	Yes	No	No	No	Yes	Yes	Yes	No	5
Roberts et al (2022)	No	Yes	No	No	Yes	No	Yes	Yes	Yes	6
Ruokolainen et al (2022)	No	Yes	No	No	No	Yes	Yes	Yes	Yes	6
Schiff et al (2021)	No	Yes	No	No	No	Yes	Yes	Yes	Yes	6
Sun et al (2022)	Yes	Yes	No	Yes	Yes	NA	Yes	Yes	Yes	9
Taylor et al (2022)	Yes	Yes	No	Yes	No	Yes	Yes	Yes	Yes	8
Wilhelm et al (2022)	Yes	Yes	Yes	Yes	No	Yes	Yes	Yes	Yes	7
Yang et al (2022)	Yes	Yes	No	Yes	Yes	Yes	Yes	Yes	Yes	9

Question codes:
Q1: Is it clear in the study what is the 'cause' and what is the 'effect' (i.e. there is no confusion about which variable comes first)?
Q2: Were the participants included in any comparisons similar?
Q3: Were the participants included in any comparisons receiving similar treatment/care, other than the exposure or intervention of interest?
Q4: Was there a control group?
Q5: Were there multiple measurements of the outcome both pre and post the intervention/exposure?
Q6: Was there a detailed description of non-response (e.g., participation/follow-up rates and reasons for non-response)?
Q7: Were the outcomes of participants included in any comparisons measured in the same way?
Q8: Were outcomes measured in a reliable way?
Q9: Was appropriate statistical analysis used?
Notes:
1. Q1: Pre-post studies without a control group were answered as "no".
2. Q3: The ideal answer is "no" (1 point). If the regression has considered other e-cigarette policies implemented in the study area and the timing of their implementation, we then assumed that there was no confounding of similar interventions.
3. Q6: As many data were repeated cross-sectional instead of longitudinal, Question 6 was adapted to "was there a detailed description of non-response (e.g., participation/follow-up rates and reasons for non-response)?". The answer was "yes" if the included study used secondary data from other longitudinal/repeated cross-sectional survey which had been fully described.
4. If the study clearly responded to the leading question, the respective parameter received a "yes" (1 point); except question 3 as described above. If the information provided was incomplete or not clear, the parameter received an "unclear" (0 point); if it was not possible to find the information, the parameter received a "no" (0 point).
